# Long-term outcomes of laparoscopic duodenojejunostomy for superior mesenteric artery syndrome

**DOI:** 10.1007/s00464-025-11774-6

**Published:** 2025-06-27

**Authors:** Mélissa V. Wills, Juan S. Barajas-Gamboa, Valentin Mocanu, Andrew Conner, John Brown, Gabriela Restrepo-Rodas, Sol Lee, Salvador Navarrete, Ricard Corcelles, Matthew Allemang, John Rodriguez, Andrew Strong, Jerry Dang, Matthew Kroh

**Affiliations:** 1 Cleveland Clinic Lerner College of Medicine of Case Western Reserve University, Cleveland, OH USA; 2 Digestive Disease Institute, Cleveland Clinic Abu Dhabi, Abu Dhabi, United Arab Emirates; 3 Department of General Surgery, Seoul Medical Center, Seoul, Republic of Korea

**Keywords:** Superior mesenteric artery syndrome, Laparoscopic duodenojejunostomy, Dysmotility disorder, Clinical outcomes, Nutritional support

## Abstract

**Introduction:**

Superior mesenteric artery syndrome (SMAS) is caused by partial obstruction of the duodenum between the SMA and the aorta. While laparoscopic duodenojejunostomy (DJ) shows favorable short- and intermediate-term outcomes, characterization of long-term outcomes remains limited.

**Methods and procedures:**

An IRB-approved retrospective study was conducted on patients who underwent laparoscopic DJ from January 2007 to January 2023 at our center. SMAS diagnosis was confirmed through clinical presentation and radiographic studies. Data on BMI, symptoms, and nutritional support were collected preoperatively and at last follow-up. A McNemar test was performed to compare preoperative and long-term outcomes.

**Results:**

Twenty-five patients (median age 32.1 years, IQR 23.2–45.3) with a median follow-up of 5.3 years (IQR 3.0–8.5) were identified. BMI increased from median of 19.2 kg/m^2^ (IQR 17.8–21.1) to 21.6 kg/m^2^ (IQR 18.0–23.8) at the time of last follow-up (*p* = 0.005). The number of patients dependent on enteral tube feeds and total parenteral nutrition decreased within this time frame (36.0% to 28.0%, *p* = 0.4795). Fewer patients underwent procedures for enteral access placement preoperatively than by the time of last follow-up (36.0% to 12.0%, *p* = 0.1573). Abdominal pain (*p* < 0.001), nausea (*p* < 0.001), vomiting (*p* = 0.3657), bloating (*p* = 0.2568), and weight loss (*p* < 0.001) decreased within this time frame. No significant changes were observed in antiemetic (*p* = 0.7389) or prokinetic (*p* = 0.5637) use. Seven patients (28.0%) required major surgical interventions for diagnoses of gastrointestinal motility disorders. Four of these patients (16.0%) died during the final follow-up period.

**Conclusion:**

Laparoscopic DJ shows long-term benefits for SMAS, including improved BMI, reduced supplemental feeding dependence, and significant symptom relief, particularly for pain, nausea, and vomiting. However, a significant proportion of patients with concurrent gastrointestinal motility disorders had worse outcomes, required additional surgical interventions, and experienced higher mortality rates, underscoring the importance of both thorough preoperative evaluation and vigilant longitudinal follow-up for this complex patient population.

Superior mesenteric artery (SMA) syndrome is a rare condition characterized by compression of the third segment of the duodenum between the SMA and the aorta. This compression leads to obstructive symptoms of chronic partial small bowel obstruction, including postprandial epigastric pain, nausea, vomiting, early satiety, and significant weight loss, potentially leading to malnutrition [1]. The pathophysiology involves a decreased aorto-mesenteric angle, typically due to conditions that reduce the mesenteric fat pad, which normally serves to maintain the angle and patency of the duodenum [2]. This can be precipitated by various factors such as trauma, malignancy, eating disorders, or other etiologies of weight loss [3].

SMA syndrome is an uncommon condition, with an estimated incidence of 0.1 to 0.3% in the general population, though it may be higher due to underdiagnosis [4]. Initial management focuses on conservative measures, including symptom control and nutritional support to prevent worsening malnutrition, increase visceral fat stores, and restore the aorto-mesenteric angle to a normal range, typically of 28 to 65º. When nonoperative management is inadequate, surgical intervention may become necessary. Several operative strategies exist, but laparoscopic duodenojejunostomy (DJ) has emerged as a preferred surgical approach [1]. It involves creating a side-to-side anastomosis between the proximal third of portion of the duodenum and the jejunum to provide an alternate drainage pathway, rather than through the obstructed duodenal segment [5]. Short-term follow-up studies have demonstrated that the laparoscopic DJ is a safe procedure with minimal perioperative complications and high success rates of 79 to 92% for symptom resolution, particularly regarding postprandial pain, nausea, and vomiting [5]. Hospital stays average 4–5 days, and early complications are primarily limited to rare cases of prolonged ileus or temporary feeding intolerance [6]. Our institution previously reported an intermediate-term analysis and revealed that despite initial improvement in all patients, only 33% (6/18) reported sustained symptomatic improvement or resolution at two-year follow-up, with certain patients developing concurrent motility disorders [7]. This notable decrease in efficacy raises important questions about the durability of surgical intervention for SMA syndrome and highlights the need for extended follow-up studies to identify predictors of surgical success beyond the intermediate timeframe.

The aim of this study was to evaluate the long-term efficacy of laparoscopic duodenojejunostomy for SMA syndrome by assessing symptom resolution, medication requirements, and nutritional support needs at both early and extended follow-up intervals. We sought to better understand the durability of this surgical intervention and identify factors that may influence long-term outcomes in this challenging disease process.

## Methods

### Study design and patient selection

**Fig. 1:**
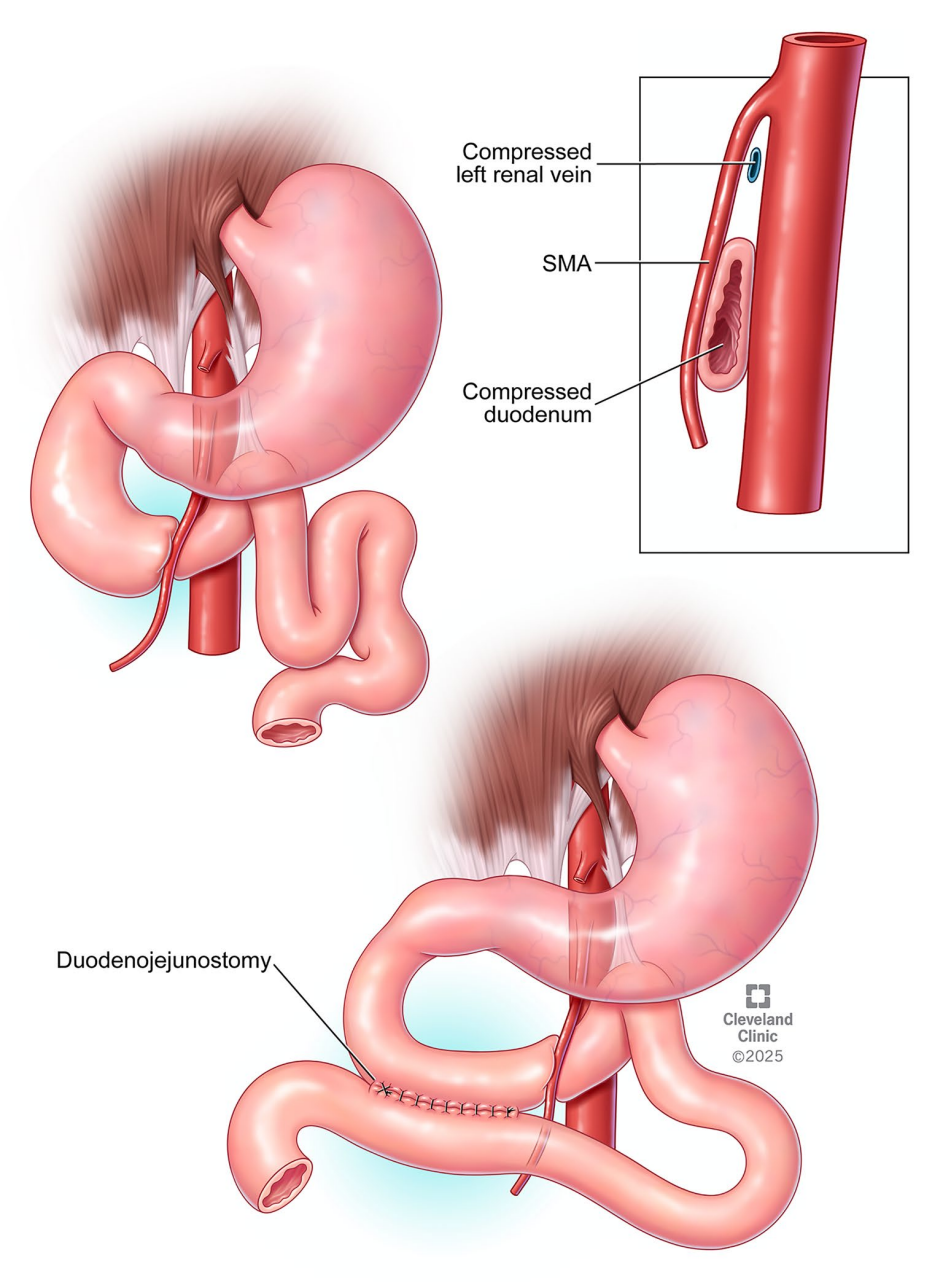
Anatomical illustration of Superior Mesenteric Artery (SMA) syndrome pathophysiology (top) showing compression of the duodenum and left renal vein, with corresponding laparoscopic duodenojejunostomy procedure (bottom) for definitive treatment

A retrospective review was conducted of all patients who underwent laparoscopic duodenojejunostomy for a diagnosis of SMA syndrome at our tertiary care center between January 2007 and January 2023. Patients sixteen years of age or older that had a follow-up of greater than two years were included. The diagnosis of SMA syndrome was confirmed by at least one of the following imaging modalities: computed tomography angiography (CTA) demonstrating narrowed aortomesenteric angle from normal range of 28–65°, ultrasound (US) with color Doppler indicating a narrowed angle with altered blood flow, or upper gastrointestinal (UGI) series demonstrating proximal duodenal dilation and obstruction at the third segment, in addition to consistent symptoms [8, 9] (Fig. 1). Patients with incomplete medical records prior to surgery, or other prior duodenal surgery, were excluded. The study protocol was approved by our Institutional Review Board.

### Laparoscopic duodenojejunostomy technique

A standardized laparoscopic duodenojejunostomy technique is performed using a 4-port approach. After establishing pneumoperitoneum, ports are placed in the umbilical region (10-12 mm), right upper quadrant (5 mm), left upper quadrant (5-10 mm), and left lower quadrant (5 mm). The transverse mesocolon is elevated to identify the duodeno-jejunal junction, confirmed by its relation to the inferior mesenteric vein. The transverse mesocolon is opened to the right of the superior mesenteric artery and a partial Kocher maneuver is performed to mobilize the duodenum in descending and proximal transverse portions. A loop of jejunum approximately 15 cm from the ligament of Treitz is selected and positioned adjacent to the second and third portions of the duodenum, proximal to the SMA crossing. Stay sutures secure the alignment, and enterotomies are created along the antimesenteric borders. A stapled side-to-side anastomosis is created using a 45 mm laparoscopic stapler, and the common enterotomy is closed with running absorbable sutures (Fig. 1). A crotch stitch reinforces the distal anastomotic margin. An endoscopic leak test is routinely performed. If indicated, additional enteral access procedures, such as percutaneous endoscopic gastrostomy (PEG), PEG with jejunal extension, and jejunostomy tubes can be performed during the same operation, though are not routinely combined in our practice [7, 10]**.**

### Data collection and follow-up assessment

Patient data were extracted from electronic medical records, including demographics, body mass index (BMI), comorbidities, prior surgical and medical history, and presenting symptoms. The symptoms assessed included abdominal pain, nausea, vomiting, weight loss, and bloating. We collected data on the use of antiemetics (including ondansetron, promethazine, and prochlorperazine, among others) and prokinetics (including metoclopramide and erythromycin). Nutritional support requirements, such as enteral tube feeding and total parenteral nutrition, were also recorded. Postoperative outcomes were evaluated at two time points: two-year follow-up and final follow-up visit. At each time point, we assessed for new or worsening symptoms, medication requirements, nutritional support needs, and subsequent procedures through review of clinical documentation. Symptom persistence or worsening was defined as patient-reported symptoms of similar or greater severity compared to prior evaluation as documented in clinical notes.

### Statistical analysis

Categorical variables are presented as frequencies and percentages. Continuous variables are presented as median with interquartile range (IQR). Statistical comparisons between preoperative, and final follow-up categorical data were performed using a McNemar test. Statistical comparisons of body mass index were performed using a T-test. A *p*-value < 0.05 was considered statistically significant. All statistical analyses were performed using STATA 17 statistical software (StataCorp, College Station, TX, USA).

## Results

Twenty-five patients underwent laparoscopic duodenojejunostomy between January 2007 and 2023 for SMA syndrome. The median age at surgery was 32.1 years (IQR 23.2–45.3), and most patients were female (18/25, 72.0%). The median length of long-term follow-up was 5.3 years (IQR 3.0–8.5). By the time of surgical evaluation, patients had lost a median of 9.3 kg (IQR 4.9–13.6) from their baseline weight, and the median preoperative BMI was 19.2 kg/m^2^ (IQR 17.8–21.1). The median BMI two years after surgery was 19.3 kg/m^2^ (IQR 18.3–23.2). The median BMI by last follow-up was 21.6 kg/m^2^ (IQR 18.0–23.8), which was significantly increased from last preoperative (*p* = 0.005). Comorbid gastrointestinal conditions assessed preoperatively included gastroparesis (10/25, 40.0%), gastroesophageal reflux disease (12/25, 48.0%), and chronic constipation (13/25, 52.0%), including a subset with idiopathic or slow transit constipation. A significant proportion of patients had diagnoses of Axis 1 disorders including generalized anxiety disorder, major depressive disorder, and bipolar disorder (15/25, 60.0%), and many patients (17/25, 68.0%) were taking at least one psychiatric medication. Four (16.0%) patients had a clinical diagnosis of an eating disorder, including anorexia and bulimia nervosa. Prior abdominal surgery was reported in 60.0% (15/25) of patients (Table 1).

**Table 1:** Demographics and baseline characteristics of patients

Characteristic	Count (%) or Median (IQR)
Age at time of surgery (years)	32.1 (23.2–45.3)
Female	18 (72.0)
Median preoperative weight loss (kg)	9.3 (4.9–13.6)
Median preoperative BMI (kg/m^2^) ± SD	19.2 (17.8–21.1)
Mood disorder	15 (60.0)
Taking ≥ 1 psychiatric medications	17 (68.0)
Eating disorder	4 (16.0)
Prior abdominal surgeries	15 (60.0)
Gastroparesis	10 (40.0)
Gastroesophageal reflux disease	12 (48.0)
Chronic constipation	13 (52.0)

Categorical data are presented as number (%) and continuous data are presented as median (interquartile range, *IQR*)

*SD* Standard deviation, *BMI* Body mass index

The median length of stay after laparoscopic duodenojejunostomy was 5.8 days (IQR 2.3–7.0). No immediate perioperative mortality was noted. Two patients had a conversion to exploratory laparotomy due to significant intra-abdominal adhesions or shortened mesentery; one patient had a re-exploration for bleeding, and one patient underwent a negative diagnostic laparoscopy for postoperative abdominal pain.

**Fig. 2:**
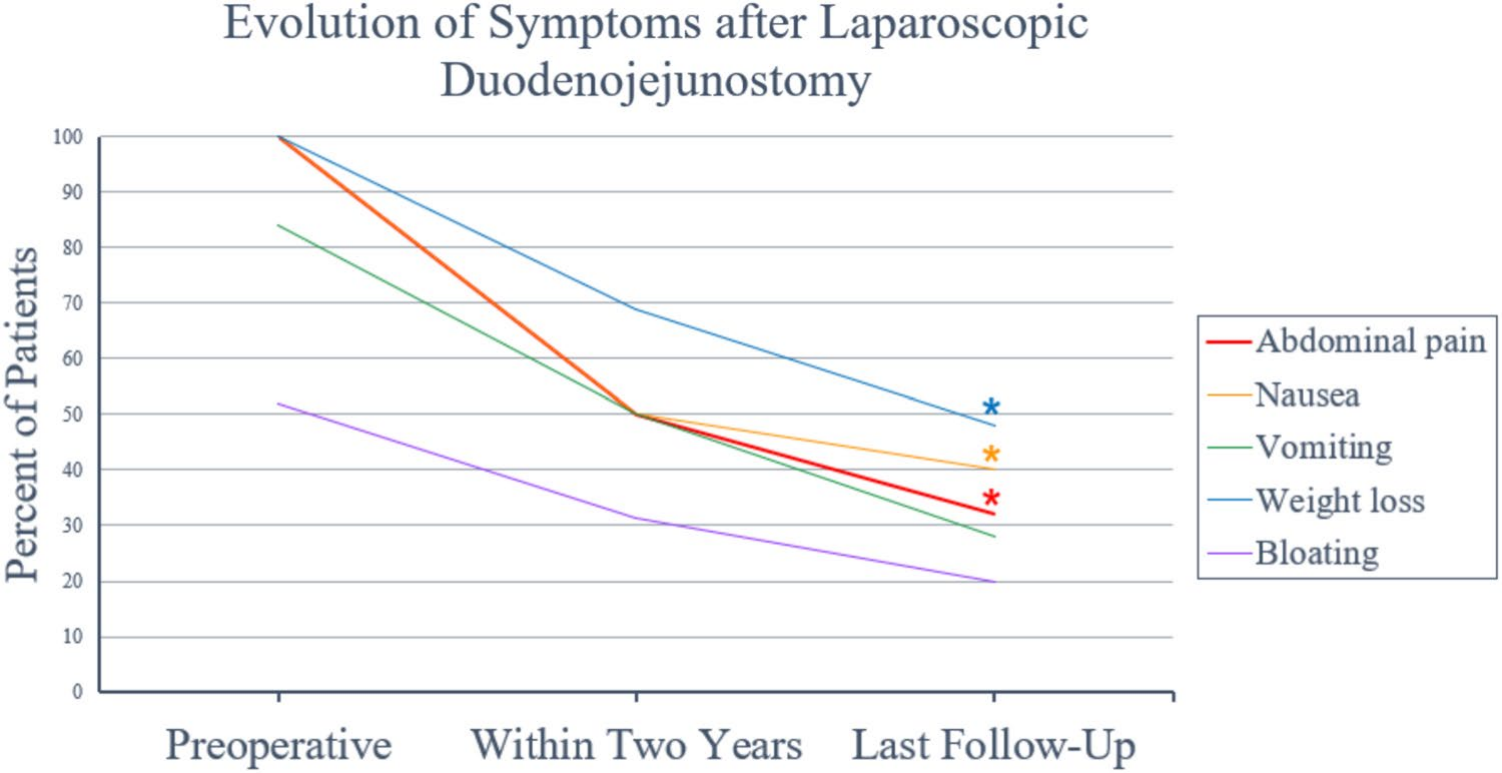
Evolution of symptoms after laparoscopic duodenojejunostomy

Preoperatively, all patients endorsed abdominal pain, nausea, and weight loss (25/25, 100%). Other presenting symptoms included vomiting in 21/25 (84.0%) patients and bloating in 13/25 (52.0%) patients. At two-year follow-up (median 24 months, IQR 22.0–29.0), the following symptoms were reported as persistent or worsening: abdominal pain (8/16, 50.0%), nausea (8/16, 50.0%), vomiting (8/16, 50.0%), weight loss (11/16, 68.8%), and bloating (5/16, 31.3%). By final follow-up (median 5.3 years, IQR 3.0–8.5), the persistent or worsening symptoms were as follows: abdominal pain (8/25, 32.0%), nausea (10/25, 40.0%), vomiting (7/25, 28.0%), weight loss (12/25, 40.0%), and bloating (5/25, 20.0%). Between preoperative and assessment at last follow-up, there was a decrease in the number of patients reporting abdominal pain (*p* < 0.001), nausea (*p* < 0.001), vomiting (*p* = 0.3657), weight loss (*p* < 0.001), and bloating (*p* = 0.2560). Patients with missing symptom data at the two-year assessment (*N* = 9) were excluded from the two-year assessment but were included in the final follow-up analysis. *P-*values represent the change from preoperative assessment to last follow-up. (Fig. 2, Table 2).

**Table 2:** Clinical features and outcomes: from preoperative assessment through two-year and final follow-up

Characteristic	Preoperative	At 2-year follow-up^a^	At last follow-up	*P-*value
Time between surgery and follow-up (years)	–	2.0 (1.8–2.4)	5.3 (3.0–8.5)	*–*
Median BMI (kg/m^2^) (IQR)	19.2 (17.8–21.1)	19.3 (18.3–23.2)	21.6 (18.0–23.8)	0.005
Symptoms				
Abdominal pain, *N* (%)	25 (100)	8 (50.0)	8 (32.0)	< 0.001
Nausea, *N* (%)	25 (100)	8 (50.0)	10 (40.0)	< 0.001
Vomiting, *N* (%)	21 (84.0)	8 (50.0)	7 (28.0)	0.3657
Weight loss, *N* (%)	25 (100)	11 (68.8)	12 (48.0)	< 0.001
Bloating, *N* (%)	13 (52.0)	5 (31.3)	5 (20.0)	0.2560
Medication requirements				
Antiemetics, *N* (%)	16 (64.0)	10 (62.5)	15 (60.0)	0.7389
Prokinetics, *N* (%)	5 (16.0)	1 (6.3)	3 (12.0)	0.5637

Categorical data are presented as number (%) and continuous data are presented as median (IQR)

*BMI* body mass index, *PEG p*ercutaneous endoscopic gastrostomy tube

^a^Nine patients did not have symptom or medication data at two-year follow-up, therefore *N* = 16

Preoperatively, 16/25 (64.0%) patients were taking one or more antiemetic medications. While this decreased to 10/16 (62.5%) patients at two-year follow-up, by final follow-up 15/25 (60.0%) patients were taking antiemetic medications (*p* = 0.7389). Prokinetic medication use was documented in 5/25 (16.0%) patients preoperatively, decreased to 1/16 (6.3%) patients at two-year follow-up, and was present in 3/25 (12.0%) patients at final follow-up (*p* = 0.5637). (Table 2).

Procedural interventions and nutritional support requirements were also tracked throughout the study period. One (4.0%) patient underwent celiac plexus block preoperatively, four (16.0%) between surgery and two-year follow-up, and one (4.0%) by last follow-up. One (4.0%) patient underwent intrapyloric botulinum toxin injection by two-year follow-up. Nine patients (36.0%) underwent enteral feeding access placement with either PEG tube, J-tube, PEG-J, or nasoduodenal tube before surgery, 5 (20.0%) by two-year follow-up, and 3 (12.0%) by last follow-up (*p* = 0.1573). The proportion of patients requiring enteral tube feeding decreased from 28.0% (7/25) preoperatively to 24.0% (6/25) at two-year follow-up and remained stable at 24.0% (6/25) at final assessment. Parenteral nutrition utilization was 24.0% (6/25) preoperatively, 20.0% (5/25) at two years, and 24.0% (6/25) at final follow-up. Supplemental nutrition requirements either with tube feeds or parenteral nutrition were documented in 9 (36.0%) patients preoperatively, 6 (28.6%) by two-year follow-up, and 7 (28.0%) at last follow-up (*p* = 0.4795) (Table 3).

**Table 3:** Procedures and nutritional support requirements through follow-up periods

Characteristic *N*, (%)	Preoperative	At 2-year follow-up	At last follow-up	*P-*value
Celiac plexus block	1 (4.0)	4 (16.0)	1 (4.0)	–
Enteral access placement	9 (36.0)	5 (20.0)	3 (12.0)	0.1573
PEG tube	4 (16.0)	4 (16.0)	1 (4.0)	–
J-tube	1 (4.0)	2 (8.0)	1 (4.0)	–
PEG-jet	2 (8.0)	1 (4.0)	1 (4.0)	–
Nasoduodenal tube	6 (24.0)	0 (0.0)	0 (0.0)	
Intrapyloric botulinum toxin injection	0 (0.0)	1 (4.0)	0 (0.0)	–
Supplemental nutrition dependence^a^	9 (36.0)	6 (28.6)	7 (28.0)	0.4795
Enteral tube feeds	7 (28.0)	6 (24.0)	6 (24.0)	–
TPN use	6 (24.0)	5 (20.0)	6 (24.0)	–
Deceased	–	–	4 (16.0)	–

Categorical data are presented as number (%) and continuous data are presented as median (IQR)

*BMI* body mass index, *PEG* percutaneous endoscopic gastrostomy tube

^a^Some patients required both enteral tube feeds and TPN simultaneously, which is why the sum of the individuals receiving nutritional support methods exceeds the total number of patients receiving supplemental nutrition

Seven patients required additional intra-abdominal procedures by the time of last follow-up. Patients underwent median arcuate ligament release (*N* = 1, 4.0%), intestinal transplant (*N* = 2, 8.0%) and partial or total abdominal colectomy (*N* = 3, 12.0%) for intestinal dysmotility, and takedown of duodenojejunostomy with pyloromyotomy for continued symptoms and diagnosis of gastroparesis (*N* = 1, 4.0%). Four (16.0%) of these patients were deceased by time of long-term follow-up. Two patients died from complications of intestinal transplant, one from malnutrition secondary to chronic abdominal pain, and one of unknown causes. The mean length of time between laparoscopic duodenojejunostomy and death was 6.9 ± 1.6 years.

## Discussion

In this study of 25 patients who underwent laparoscopic duodenojejunostomy for superior mesenteric artery syndrome with a median follow-up of 5.3 years (IQR 3.0–8.5), we observed significant improvement in BMI (from 19.2 to 21.6 kg/m^2^) and key symptoms, with resolution rates of 68% for abdominal pain, 60% for nausea, and 52% for weight loss (all *p* < 0.001). Despite these improvements, 28% of patients remained dependent on supplemental nutrition, 28% required subsequent major operations (including intestinal transplantation, colectomy, pyloromyotomy, and MALS release), and 16% died during the follow-up period. While symptom improvement remained stable between intermediate- and long-term follow-up, suggesting durability when successful, the high rate of nutritional support dependence and mortality underscores the complex nature of this condition and the importance of careful patient selection for surgical intervention.

Superior mesenteric artery syndrome, also known as Wilkie’s Disease, is defined by compression of the third portion of the duodenum between the SMA and the aorta (Fig. 1) [7]. Given its nonspecific presentation—postprandial epigastric pain, vomiting, and progressive weight loss—SMA syndrome often eludes early detection, frequently discovered only after more common etiologies have been ruled out [11, 12]. This delay in diagnosis is particularly consequential as SMA syndrome can be frequently precipitated by conditions that cause rapid weight loss, such as HIV, malignancy, eating disorders, trauma, and amyotrophic lateral sclerosis [1, 3, 13].The resulting nutritional compromise not only exacerbates the underlying pathology but also diminishes the patients’ tolerance for essential interventions, including enteral feeding access and definitive surgical correction. Thus, though the disease is rare, maintaining a high index of suspicion for SMA syndrome is crucial, particularly in patients with predisposing conditions or progressive nutritional decline.

Our cohort demonstrated a strong female predominance (72.0%), consistent with established SMA syndrome demographics—Ganss et al*.* reported 71.8%, while Biank and Werlin found 64% female patient proportion in their respective cohorts [12, 14]. This female predisposition primarily stems from sex-specific anatomic characteristics: female patients typically have lower baseline visceral fat content, leading to reduced mesenteric fat pad volume and narrower aortomesenteric angles, as demonstrated by Ozbulbul et al [15]. These anatomic considerations provide context for the demographic patterns observed in our study, including the presence of psychiatric comorbidities (60.0%) and eating disorders (16.0%). These findings align with prior data by Lee et al., who previously reported these comorbidities in 21.3% of patients in their cohort [16]. The relationship between SMA syndrome and these conditions appears to be multifaceted and bidirectional- the chronic pain, nutritional challenges, and symptoms associated with the disease may contribute to psychological distress, while conditions linked to weight loss can simultaneously exacerbate SMA syndrome by further diminishing the protective mesenteric fat pad. As a result, patients can present in a nutritionally and psychiatrically vulnerable state, which underscores the importance of comprehensive preoperative nutritional optimization and appropriate management of all comorbidities to maximize the likelihood of successful surgical intervention [13].

The frequency of subsequent intra-abdominal procedures and mortality after laparoscopic duodenojejunostomy in our cohort warrants careful consideration and represents a key finding of this study. Despite radiographic evidence of SMA syndrome in all patients, seven required additional surgical interventions. However, the majority of these interventions were targeting to other gastrointestinal pathologies, underscoring the significant burden of concurrent disease—particularly with gastric and intestinal dysmotility. Three patients underwent colectomy and two underwent intestinal transplant for intestinal or global gut dysmotility despite resolution of duodenal obstruction after laparoscopic duodenojejunostomy. One patient required median arcuate ligament release for median arcuate ligament syndrome, pointing to a potential predisposition to multiple vascular compression syndromes. Finally, one patient had gastroparesis eventually requiring duodenojejunostomy takedown and pyloromyotomy, highlighting the diagnostic challenges in distinguishing mechanical obstruction from functional dysmotility.

These findings suggest that SMA syndrome can be comorbid with other significant gastrointestinal pathologies– a finding that complicates an already challenging diagnostic picture [7]. While maintaining a high index of suspicion for SMA syndrome remains crucial, these observations underscore the importance of judicious patient selection when pursuing surgical intervention. Our results also suggest potential gaps in our current diagnostic approach, which relies heavily on anatomical criteria while possibly overlooking concurrent functional disorders. They highlight the need for comprehensive preoperative evaluation, including assessment of gastrointestinal motility, which may help identify patients with complex pathophysiology who may not benefit from duodenojejunostomy alone. Future research is needed to develop a standardized patient-reported outcome measure to distinguish SMA syndrome from conditions with overlapping symptomatology, as their respective surgical interventions differ substantially and carry significant morbidity in this vulnerable patient population.

Additionally, our 16% mortality rate is notably higher than previous series—Ganss et al*.* had no deaths among 39 patients at 47 months, Sun et al*.* reported no deaths in 14 patients at 20 months, and Chang et al*.* documented no deaths in 18 patients at 26 months [6, 7, 14]. While our extended follow-up period and complex patient population may partially explain this difference, these outcomes warrant careful analysis and underscore the significant co-morbidity of conditions that patients may have. No deaths occurred in the peri-procedural period. However, two deaths occurred as complications of intestinal transplantation, one from malnutrition, and one from unknown causes. The occurrence of deaths specifically among patients requiring subsequent major interventions suggests that persistent post-operative symptoms may indicate a more complex underlying disease process. These findings highlight the importance of close post-operative surveillance and early recognition of treatment failure.

Through review of electronic medical records, we also evaluated the evolution of symptoms before surgery, at two-year follow-up, and at last follow-up. Our outcomes suggest that for certain patients, laparoscopic duodenojejunostomy can provide sustained symptomatic relief. We observed meaningful symptom resolution of abdominal pain, nausea, vomiting, weight loss, and bloating, at two-year follow-up that remained largely stable through final follow-up, suggesting long-term effectiveness of the procedure when successful. This finding is significant given ambiguity in the literature regarding long-term stability of outcomes. In a series of 14 patients, we had previously demonstrated that 92% of patients had improvement or elimination of symptoms immediately after surgery, and that that 79% maintained symptom improvement by a median follow-up of 20 months [6]. Our group then found that though 14 of 18 patients reported initial symptom improvement after surgery, only six maintained symptomatic improvement by median follow-up of 26 months [7]. Jain et al*.* demonstrated that 19 of 22 patients experienced no symptom recurrence at mean follow-up of 41.2 months [17]. Our data, with a median follow-up of 5.3 years, demonstrates that when initial symptomatic improvement is achieved, it tends to persist beyond this intermediate window, contributing important evidence regarding the procedure's long-term durability. It is worth noting that despite these symptomatic improvements, the number of patients taking antiemetics and prokinetics did not significantly change after surgery. This persistent medication requirement despite reported symptom improvement suggests that patients may experience residual discomfort below reporting thresholds, or that clinicians maintain pharmacotherapy prophylactically following initial therapeutic response.

The high rate of nutritional support dependence at final follow-up warrants careful consideration. Of the seven patients requiring supplemental nutrition a final follow-up, six experienced major adverse events including mortality or the need for major intra-abdominal operations. This observation suggests that when laparoscopic duodenojejunostomy successfully addresses an isolated underlying SMA syndrome pathology, patients typically achieve independence from supplemental feeding. Conversely, persistent dependence on nutritional support may signal either incomplete resolution of the duodenal obstruction or presence of additional gastrointestinal pathologies requiring separate intervention. Based on these findings, we propose that continued nutritional support dependence at six to twelve months postoperatively should prompt re-evaluation for concurrent gastrointestinal disorders or technical failure of the bypass.

Despite being the largest series with greater than two-year follow-up, this study has several limitations. As a single-institution retrospective review, our findings may not be generalizable to all populations with SMA syndrome. The relatively small sample size of 25 patients, while comparable to existing literature given the rarity of the condition, limits our ability to perform meaningful subgroup analyses or identify statistically significant predictors of surgical success. Additionally, our two-year follow-up data is incomplete, with 9 of 25 patients (36.0%) lacking comprehensive symptom documentation. While other data points such as BMI, procedural interventions, and nutritional support were more completely captured, the missing symptom data introduces potential bias in the intermediate-term analysis. The retrospective nature of symptom assessment through chart review, rather than standardized patient-reported outcome measures, may not fully capture the nuanced changes in patients' clinical status over time. We also acknowledge potential selection bias, as patients with more severe or persistent symptoms may be more likely to continue following-up, potentially overestimating symptom persistence rates. Future multi-institutional prospective studies and the creation of patient-reported outcome measures are needed to better characterize long-term outcomes and identify optimal candidates for surgical intervention.

## Conclusion

This long-term analysis of laparoscopic duodenojejunostomy for SMA syndrome demonstrates that while surgical intervention can provide durable symptom relief in select patients, the high rates of nutritional support dependence (28%), subsequent major operations (28%), and mortality (16%) highlight the complexity of patient selection. The findings suggest SMA syndrome often coexists with other gastrointestinal pathologies affecting outcomes. Based on our findings, we recommend comprehensive preoperative evaluation including gastric emptying studies and motility testing to identify patients with concurrent motility disorders who might benefit from alternative or additional interventions. Persistent nutritional support dependence postoperatively should prompt investigation for additional gastrointestinal pathologies. Future prospective, multi-institutional studies using standardized measures are needed to identify optimal surgical candidates and refine treatment algorithms for this challenging disease.
